# Report of Eruptive Fevers Treated in General Hospitals, Department of Virginia, from October 1, 1862, to January 31st, 1864

**Published:** 1864-03

**Authors:** Wm. A. Carrington

**Affiliations:** Medical Director.


ART. IV.—*Report of Eruptive Fevers treated in General Hospitals, Department of Virginia, from October* 1, 1862, *to January* 31*st*, 1864. Consolidated by Surgeon WM. A. CARRINGTON, Medical Director.

| MONTH. | ERYSIPELAS. | | RUBEOLA. | | SCARLATINA. | | VARIOLA. | | VARIOLOIDES. | | ALL OTHER, &C. |
|---|---|---|---|---|---|---|---|---|---|---|---|
| | Cases. | Deaths. | Cases. | Deaths. | Cases. | Deaths. | Cases. | Deaths. | Cases. | Deaths. | Cases. |
| 1862—October | 38 | 5 | 337 | 3 | 4 | 0 | 12 | 1 | 1 | 0 | 17 |
| November | 58 | 3 | 298 | 8 | 2 | 0 | 161 | 30 | 54 | 0 | 10 |
| December | 68 | 5 | 158 | 3 | 1 | 0 | 424 | 145 | 213 | 5 | 17 |
| 1863—January | 118 | 15 | 146 | 0 | 5 | 3 | 438 | 221 | 169 | 9 | 5 |
| February | 99 | 10 | 92 | 7 | 1 | 1 | 187 | 137 | 87 | 5 | 27 |
| March | 70 | 11 | 136 | 9 | 34 | 0 | 133 | 57 | 57 | 4 | 19 |
| April | 127 | 9 | 80 | 8 | 4 | 0 | 192 | 62 | 120 | 1 | 59 |
| May | 200 | 13 | 132 | 1 | 2 | 0 | 66 | 39 | 41 | 1 | 123 |
| June | 165 | 14 | 91 | 5 | 3 | 1 | 110 | 24 | 60 | 1 | 67 |
| July | 86 | 6 | 48 | 1 | 2 | 0 | 43 | 30 | 68 | 2 | 35 |
| August | 74 | 5 | 51 | 1 | 0 | 0 | 8 | 4 | 6 | 2 | 7 |
| September | 40 | 1 | 18 | 0 | 0 | 0 | 0 | 0 | 1 | 0 | 15 |
| October | 50 | 6 | 6 | 1 | 0 | 0 | 13 | 2 | 9 | 0 | 19 |
| November | 59 | 2 | 33 | 1 | 0 | 0 | 100 | 13 | 27 | 0 | 32 |
| December | 72 | 3 | 87 | 1 | 0 | 0 | 403 | 114 | 96 | 0 | 5 |
| 1864—January | 62 | 0 | 494 | 2 | 0 | 0 | 223 | 141 | 187 | 9 | 86 |
| Totals | 1,386 | 108 | 2,207 | 46 | 58 | 5 | 2,513 | 1,020 | 1,196 | 39 | 543 |
| Per centum of Deaths | | 7.79 | | 2 09 | | 8.62 | | 40.58 | | 3.26 | |

Per centum of eruptive fevers (7,903) to total number of cases treated (178.586)=4.42.
Per centum of deaths from eruptive fevers (1,218) to total number of deaths (10.961)=11.11.
Mortality in all cases treated, 6.13; in eruptive fevers, 15.41; in variolous diseases, 28.76.

Eruptive fevers constitute an important class in the reports required from medical officers of the Confederate States Army. On inspecting the records of the first division of this class, erysipelas, the uniformity in the number of cases occurring in each month is worthy of notice; while in other divisions the well known prevalence in the colder, and the diminution in the warmer seasons of the year, are well illustrated, especially in the reports of small-pox in the months of August, September, December and January. It should be considered that many of the cases reported as of this divition are traumatic, and should not properly be classified among "eruptive fevers," but as instances of "local erysipelatous inflammation."

The second division of this class is one of the most interesting to the army surgeon, from its universal prevalence among soldiers. The reports from April, 1861, to October, 1862, would show a much greater prevalence than here reported. When it is remembered that only the severer cases of this disease are sent to hospitals, the mortality appears slight.

The reports of scarlatina bear out the experience of previous observers, that it is not a disease of armies.

The sixth division, embracing all anomalous forms of eruptive fever, were probably cases of *roseola*, and were so slight that no deaths are reported as having occurred among them.

The fourth and fifth divisions of this class, comprising modifications of the same disease, will painfully attract, both by the large number of cases occurring and the rate of mortality. From actual observation and investigation at the time, I can definitely pronounce upon the origin and progress of small-pox in these hospitals. On October 18th, 1862, the first cases were brought to Richmond from Fort Delaware. Up to that time no cases had been reported here for some months, in the army or among citizens. By the 31st of October twelve cases had been reported. In carefully tracing each, it was determined that those from Fort Delaware did not disseminate the disease, being quarantined and avoided by all, but that soldiers from the Army of Northern Virginia had brought the disease to the hospitals, and, being unconscious and unsuspected, had exposed many to it before the diagnosis was made. The army had just reached the vicinity of Winchester after evacuating Maryland, subsequent to the battle of Sharpsburg There were but few cases from the army, and those had not been prisoners, nor had they seen any returned prisoners. These cases went to Charlottesville, Lynchburg and Richmond, at which points the malady spread, but much more rapidly and extensively at Richmond.

It is a fact of interest that the disease has not spread in the army. The free ventilation of the field, with the men in bivouac and without even tents, seems to have so diluted the poison that it could not reproduce the disease in others. So in general hospitals no combination of remedies, regimen or expedients have been found so much to influence the progress or termination of the disease as free ventilation. A diminution of the capacity of the hospitals from 800 to 1600 cubic feet for each patient showed sensible effects, and removal to large tents still more reduced mortality.

There are several reasons by which we may rationally account for this great excess in the mortality of small-pox over that ordinary in civic practice:

1st. The mass of the army, being from the rural population, became soldiers unprotected by vaccination. In cities where the rich and the poor are by public or private expense all vaccinated for generations, the disease assumes a modified form, *while*, in races not hereditarily protected, as among our American Indians, it assumes as malignant a form as that existing before Jenner's great discovery.

2d. The subjects of the disease were men previously debilitated by privations, fatigue and disease, and oppressed with nostalgia, and to this is now superadded the consciousness of being afflicted with a loathsome, obnoxious and dangerous disease, and the bad effects of removal from one hospital to another.

3d. *The malignity of this epidemic.*—Of this I have much evidence from my own observation; but I beg leave to quote the very forcible and apposite remarks on this subject contained in a letter of January 28th, 1863, from Dr. Albert Snead, City Physician of Richmond:

"I have had charge of the City Hospital for about ten years, and never during that time has there been so extensive prevalence of small-pox, nor has the percentage of mortality risen so high, as during this visitation. Not only among the soldiers who have been under my care, but also among the resident population, the mortality has been fearfully great. Nor have I ever in any former visitation witnessed so large a percentage of confluent cases, or so many strictly malignant cases—cases in which the experienced eye would detect evidences of a necessarily early and fatal termination. Before this visitation I have never seen a case so malignant as to terminate life in less than six days from the first appearance of the eruption, while, during this prevalence of the disease, I have seen many cases ending in less time, some within three or four days. Moreover, heretofore I have never seen a case of varioloid prove fatal, while in this visitation I have witnessed several cases terminating in death, and that, too, after the patients had apparently been doing well for days together, when suddenly they would begin to give away without manifesting any special local trouble, and, despite the use of stimulants and other sustaining treatment, gradually sink and die. I have been distressed and annoyed at these unexpected deaths, and can only account for them by supposing that the poison in this visitation is unusually malignant, overcoming the vital powers even in those cases which for a time appeared to be proceeding favorably. I have observed that the tendency to the formation of abscesses is greatly increased over former visitations—patients who have suffered moderately mild attacks, even of varioloid, scarcely escaping them. From this I infer that the poison of the visitation is with difficulty eliminated, the endermic eruption, as is usual, not accompanying it."

As extraordinary efforts have been made to protect by vaccinating, with pure and reliable virus, the army and all persons exposed to its influence; as the most scientific means have been taken to prevent the disease, and to treat it, when incurred, by every resource known to the profession, I cannot attribute the mortality to want of care or skill in the medical officers having charge of the cases.

I will, in closing, notice two untried expedients which have been lately suggested in foreign medical journals:

1st. It is asserted that by producing, in the first stage of the disease, a free pustulation on the upper part of the chest with *tartar emetic ointment*, the eruption on the face will be prevented from appearing to the same extent that it otherwise would, the prior pustulation acting as a counter-eliminative or counter-irritant. This remedy is now being fully tried in the hospitals near this city, and the result will be reported.

2d. In the United States and England recently, a notoriety has been given to the Sarracenia Purpura in the treatment of small-pox. Very contradictory reports appear in the English medical journals, but several laud its effects, stating that "the unmistakeable evidence of the efficacy of this remedy in arresting the progress of small-pox has been conspicuously manifested in many cases."

Information has been given me by a reliable physician from Baltimore that other physicians in that city were using it with the *alledged* result of arresting the disease on about the sixth or seventh day of the eruption, when it would result in desiccation by the tenth day, with little or no pitting.

This is a remedy indigenous to this continent, being found in the marshes from Canada to Florida, and known popularly as the side-saddle plant or fly-trap. The formula for its use is an infusion prepared with two ounces of the sliced-root to two pints of water, of which from four to eight fluid ounces are taken during the twenty-four hours.

This plant was known to botanists, and held in some estimation as a domestic remedy, but was first scientifically experimented with by Surgeon F. Peyre Porcher, P. A. C. S., who published an article on its medicinal and chemical properties in the "Charleston Medical Journal," in 1849. By proving it on himself and others, he found that it was a bitter tonic, and a stimulant to the stomach and the peristaltic action of the whole alimentary canal—that it promoted the renal and other glandular secretions—produced increased action of the circulatory system, with increased frequency of pulse, irregularity of the heart's action, and decided cerebral disturbance.

The chemical analysis showed a complexity in its elements, and, among others, an alkaloid resembling *cinchonia*.

In a sluggish and torpid condition of the stomach, liver, bowels and kidneys, Surgeon Porcher used it with decided effects, and reports, in "The Resources of the Southern Fields and Forests," 1863, its extensive use in South Carolina and Georgia.

Not being on the supply table of indigenous remedies, I have not been able to obtain the remedy from the Medical Purveyor, but have taken steps to procure a supply, when its value will be faithfully tested, and report made of the result.

